# Hospital Meetings, &c.

**Published:** 1899-02-04

**Authors:** 


### HOSPITAL MEETINGS, &C.

### GERMAN HOSPITAL.

THE annual general court of governors of the German Hospital was held at Winchester House, Old Broad Street, on Friday last (26th inst.), Mr. A. J. ALLEN presiding. The statement of accounts for the past year gave the ordinary income (including a balance from 1897 of £737) as £9,221, and the ordinary expenditure as £9,500. Including legacies (£1,050), special appeal fund (£4,494), sale of funded property (£2,967), and special donation from the Prince of Wales's Fund (£150), the total income was £17,934; while the total expenditure, including £4,103 on improvements, £4,000, repayment of loan; and £200, investment of legacy, was £17,805. There was an excess of expenditure over income of £120, covered by a temporary loan from bankers of £250. The report read by Mr. Hermann Gülich, the secretary and superintendent, expressed the satisfaction of the governors that they had been able to discharge the liability incurred in carrying out the sanitary improvements and other structural work. This expenditure was originally estimated to involve about £6,000, but eventually amounted to £7,461. To meet this Mr. Bruno Schröder initiated the collection of a special fund by which no less than £4,494 was raised, and the committee were thereby enabled to reinvest £1,075, part of the proceeds of the disposal of a portion of the funded property of the hospital previously sanctioned. The hospital has now 130 beds at its disposal, in addition to 17 in the convalescent home in Dalston Lane, and with the completion of the internal arrangements the greater efficiency of the institution has made itself apparent by an increase in in-patients. In the course of the year these numbered 1,913, as compared with 1,644 in 1897. The out-patients also increased, the number rising to 25,722, including those treated at the Eastern and Western Dispensaries. These increases in numbers necessitate increases in the staff, and the committee recommend the appointment of Dr. Karl Fürth as hon. assistant physician and Dr. Willi Hirt as assistant resident medical officer, as well as Dr. Richard Wunsch in place of Dr. Albert Steiner resigned, as resident medical officer. These appointments were duly confirmed by the meeting, as were also the recommendations that M. Ch. D. Bourcart, the Swiss Minister, and Baron G. von Lindenfels, the German Consul-General, be elected vice-presidents of the hospital. In moving the adoption of the report, the Chairman referred with regret to the continued decrease in the annual subscriptions, which had dwindled down from £2,200 in 1891 to £1,487, and hoped that every effort would be made to remedy this. The adoption of the report having been agreed to, the committee was re-elected, votes of thanks were passed to the president and officers, and a similar compliment to the chairman for presiding concluded the proceedings.

---

### ITALIAN HOSPITAL.

THE annual meeting of the Italian Hospital was held on Saturday last (28th inst.) at the Italian Embassy, 20, Grosvenor Square, Baron DE RENZIS, the Italian Ambassador, presiding. The accounts presented showed a total ordinary income of £2,747, but in this was included a donation of £1,500 from the trustees of the late Giovani Silvani. The total ordinary expenditure was £765, with a sum of £4 6s. for repairs under the head of extraordinary expenditure.

There was thus a balance shown of surplus of income over expenditure for the year, other than capital account, of £1,978. The report, which was read and agreed to, gave the number of out-patients treated during the year as 4,583, the in-patients, owing to the fact that the hospital was demolished for rebuilding purposes in June, numbering only 95. The council referred with great regret to the death in the latter part of the year of Comm. G. B. Ortelli, the founder of the hospital. After some considerable discussion as to the advisability of leaving the matter open for the time being it was resolved to fill the five vacancies occurring on the council, and the names proposed, including that of the widow of the late founder, were adopted. The customary votes of thanks brought the proceedings to a close.

